# Variables influencing conditioning-evoked hallucinations: overview and future applications

**DOI:** 10.1017/S0033291722002100

**Published:** 2022-10

**Authors:** Benjamin R. Fry, Dominic Roberts, Katharine N. Thakkar, Alexander W. Johnson

**Affiliations:** 1 Department of Psychology, Michigan State University, East Lansing, MI, USA; 2 Neuroscience Program, Michigan State University, East Lansing, MI, USA

**Keywords:** Psychosis, schizophrenia, animal models, translational

## Abstract

Hallucinations occur in the absence of sensory stimulation and result in vivid perceptual experiences of nonexistent events that manifest across a range of sensory modalities. Approaches from the field of experimental and cognitive psychology have leveraged the idea that associative learning experiences can evoke conditioning-induced hallucinations in both animals and humans. In this review, we describe classical and contemporary findings and highlight the variables eliciting these experiences. We also provide an overview of the neurobiological mechanisms, along with the associative and computational factors that may explain hallucinations that are generated by representation-mediated conditioning phenomena. Through the integration of animal and human research, significant advances into the psychobiology of hallucinations are possible, which may ultimately translate to more effective clinical applications.

## Introduction

Hallucinations are perceptual experiences in the absence of corresponding sensory input. They can range from simple murmurings to complex, fully-formed voices and images. They can be comforting or distressing, and they present across a range of medical and psychiatric conditions, as well as in a sizeable proportion of the normative population (Johns et al., 2004). Although hallucinations across (and within) these different populations likely represent equifinal outcomes – with different etiologies culminating in similar symptoms – establishing a common framework to understand them may prove a powerful means by which to elucidate their mechanisms and what may go awry in more serious clinical manifestations (Corlett & Schoenbaum, 2021). Drawing on a classic body of work, recent studies in the field of learning have begun to document how associative links between stimuli can give rise to instances where an evoked representation of a sensory experience is experienced as reality (Corlett & Schoenbaum, 2021; Koh & Gallagher, 2020; McDannald & Schoenbaum, 2009), otherwise known as impaired reality testing. In the context of pre-clinical associative learning studies, impaired reality testing is demonstrated when a stimulus is able to evoke perceptual processing of absent features related to a second stimulus with which it was previously paired (e.g. repeated pairing leads an auditory tone to evoke taste features of sucrose, even when the sucrose is not presented). Predating and stemming from this animal work is a body of research which has adopted associative learning approaches to understand the conditionability of perceptual experiences (i.e. conditioned hallucinations) in humans (e.g. Corlett & Powers, 2018; Ellson, 1941, 1942; Graham, 1969; Kafadar *et al*., 2020; Powers, Mathys, & Corlett, 2017; Seashore, 1895). While previous reviews (e.g. Koh and Gallagher, 2020) have focused more explicitly on preclinical models of hallucinations, this review seeks to bridge the gap between human conditioned hallucination studies, and impaired reality testing in animals, while highlighting commonalities and diverging features. Toward that end, we will examine both historical and contemporary examples of conditioned hallucinations and discuss the factors that may mediate their strength. Furthermore, we will review current neurobiological, associative learning, and computational perspectives which attempt to explain the generation of hallucinations. It is our belief that integrating human (Table 1) and animal (Table 2) research will lead to improved insights into hallucinations which may ultimately translate to clinical applications.

**Table 1.** Summary of conditioned hallucinations (CH) studies in human participants, including sample composition, modality in which the first and second (paired) stimulus was presented, any factors included to investigate moderators of the strength of conditioned hallucinations, features of the training period, how conditioned hallucinations were measured, and a summary of the findings

Reference	Sample	S1^a^	S2^b^	Moderators	Training features^c^	Measurement of CH^d^	Findings
Seashore (1895) *	Single case design	Visual	Tactile		Yes.	Presence	Across manipulations, conditioned hallucinations were reported in the presence of S1.
	*n* = 19	Auditory	Auditory		Unclear. 10	Presence	Participants reported hearing a sound in the absence of sensory stimulation on 34/60 test trials.
Kelly (1934)	*n* = 13 (college sample)	Auditory	Visual		No. 280–3000.	Presence	No participant reported seeing a color in the presence of its associated tone.
	*n* = 5 (college sample)	Auditory	Visual	Effects of mescal	No. 1000.	Presence	No participant reported seeing a color in the presence of its associated tone.
Leuba (1940; a)	*n* = 8 (college sample); single case design	Mixed	Mixed	Hypnosis	N/A	Presence and Discrimination Judgement	Hallucinatory responses were reported for auditory-tactile, auditory-visual, auditory-olfactory, tactile-visual, tactile-olfactory, visual-auditory S1-S2 pairings.
Ellson (1941)	*n* = 190 (college sample)	Visual	Auditory	Differential instructions: participants were asked to report the tone as soon as they heard it, even if their certainty was low	Yes. <60.	Presence	The experimental group reported more conditioned hallucinations than controls. Changing task instructions did not reliably increase the number of conditioned hallucinations.
Ellson (1942)	*n* = 30 (college sample)	Visual	Auditory	Effect of extinction trials on conditioned hallucination Effect of telling participant's the tone would not be present in subsequent trials	Yes. 60	Presence	Conditioned hallucinations persisted through extinction trials. Participants continued reporting a tone in the presence of a light, even when informed that it would not be present.
Howells (1944)	*n* = 8 (college sample)	Auditory	Visual	Motivation: participants were told that mistakes on this task would incur a financial penalty and risk termination of their job. There was no control group.	Yes. 5000	Both	Participants were four times more likely to report the wrong color, in the presence of its associated tone, on test trials. When asked to adjust the color of a screen, in the presence of the tone associated with green, participants overestimated the amount of red required to make the screen appear white.
Corn-Becker et al. (1949)	*n* = 93 (college sample)	Visual	Tactile		No. 14.	Presence	Participants had greater skin concordance responses to the word ‘electric shock’ after learning to associate words with an outcome. Corroborated by verbal self-reports of tingling palms or itching.
Leuba and Dunlap (1951)	*n* = 4 (college sample) who showed evidence of being easily hypnotized; single case design	Mixed	Mixed	Hypnosis	Unclear.	Presence	When participants were asked to imagine experiencing S1, they tended to report perceptions of S2. This effect was observed for auditory-tactile, tactile-olfactory, olfactory-visual pairing, auditory-visual.
Naruse and Obonai (1953)	*n* = 32 (ages 12–15)	Auditory	Visual	Hypnosis	No. 4-5.	Presence	Participants reported vague images in the presence of the auditory stimulus. Pairing two images with two auditory stimuli resulted in participants seeing some combination of both images in the presence of both sounds.
Hefferline and Perera (1963)	*n* = 1	Tactile	Auditory		Yes. 500-2000.	Presence	The participant reported hearing a tone each time their finger twitched, even in trials in which the tone was no longer present.
Fishkin (1969)	*n* = 64 (college sample)	Auditory	Visual	Whether S1 was present only during presentation of S2 or S1 was presented several seconds before S2.	Yes. 25.	Presence	Experimental groups reported seeing the test figure (a stationary circle or spiral) getting bigger or moving towards them in the presence of a tone compared to the control group. This effect was weakest in the condition where the delay between S1 and S2 onset was longest.
Graham (1969)	*n* = 11 (high school sample) who showed evidence of being easily hypnotized	Auditory	Visual	Hypnosis	Yes. 10.	Presence	Participants who received a post-hypnotic suggestion to hallucinate reported significantly more hallucinations than did participants who were not hypnotized.
	*n* = 12 (high school sample) who showed evidence of being easily hypnotized	Auditory	Visual	Suggestion: participants were told to response as if they were hypnotized	Yes. 10.	Presence	Participants in this experimental group reported significantly fewer conditioned contrast hallucinations than did the hypnotized participants in experiment 1
Warburton et al. (1985)	*n* = 21 (college sample)	Visual	Auditory	Effect of scopolamine	Yes. 80.	Presence + Absence.	Placebo condition: 29% of participants reported at least one conditioned hallucination. Scopolamine: 95% of participants reported at least one conditioned hallucination. Dose-response effect of scopolamine: higher doses produced more conditioned hallucinations.
McIntosh et al. (1998)	*n* = 10 subjects	Auditory	Visual		Yes. 200 total.	N/A	Activity in the dorsal occipital cortex progressively increased during training such that the presence of the tone alone was able to elicit similar activity in this region as that of a visual stimulus. Correlations between occipital and prefrontal cortex became excitatory as learning progressed.
Kot and Serper (2002)	*n* = 30 persons with schizophrenia with (*n* = 15) and without (*n* = 15) history of auditory-verbal hallucinations	Visual	Auditory	Previous experience of auditory hallucinations	Yes. 60.	Presence + Absence.	Prior experience of hallucinations resulted in significantly more hallucinations. Conditioned hallucinations were harder to extinguish in people who had prior experience of hallucinations.
Powers et al. (2017)	*n* = 60 with history of psychosis and auditory-verbal hallucinations (AVHs; *n* = 15), with history of psychosis without AVH history (*n* = 15); with history of AVH but no psychosis (*n* = 15), and with no history of mental illness or AVH (*n* = 15)	Visual	Auditory	Previous experience of psychosis and AVHs	Yes.	Presence + Confidence rating	Persons with previous experience of hallucinations endorsed significantly more conditioned hallucinations. Confidence and frequency of conditioned hallucinations correlated with hallucination severity outside of experimental conditions. No group differences in brain activity were found during conditioned hallucinations.
Kafadar et al. (2020)	*n* = 36, including individuals at high-risk for psychosis (*n* = 19) and controls (*n* = 17)	Visual	Auditory	Psychosis risk	Yes.	Presence + Confidence rating	Individuals at high risk for psychosis were more likely to report conditioned hallucinations. Conditioned hallucinations correlated with clinical measures of hallucinations. Individuals at high risk of psychosis were less able to detect changing contingencies between the light and tone across trials than controls.

a Modality in which the first stimulus (S1) was presented. Mixed indicates that the authors used more than one S1 modality.

b Modality in which the second, paired stimulus (S2) was presented. Mixed indicates that the authors used more than one S2 modality.

c Indicates whether participants had to respond to S2 in the presence of S1 during training, or if they passively observed the relationship. This column also indicates the number of training trials, when that information was reported.

d Indicates whether conditioned hallucinations were measured by asking participants to report the presence of a stimulus (present/not present) or by requiring participants to make a discrimination judgement about S2 features.

*Author also reports on a series of pilot tests in which gustatory, tactile, and olfactory percepts were elicited through increasing a participant's expectation of the delivery of these stimuli.

**Table 2.** Summary of representation mediated learning and performance studies including model system, behavioral and physiological manipulations, primary outcome measures and overall findings

Model	Manipulation(s)	Behavioral Paradigm	Dependent Measure(s)	Outcome	Authors Year
Rat (Sprague Dawley)	Pavlovian conditioning stimulus modality (LiCl v. electrical shock)	RMTA	Overall intake, liquid sucrose; sucrose pellet	Administering LiCl but *not* electrical shock in the presence of a cue previously associated with sucrose induces RMTA	Holland (1981)
Rat (Sprague Dawley)	Minimal (16 CS/US pairings) v. extensive (64 CS/US pairings) Pavlovian conditioning	RMTA	Overall intake, liquid sucrose	Minimal Pavlovian conditioning is sufficient for RMTA; extensive conditioning prevents RMTA	Holland (1998)
Rat (Sprague Dawley)	Minimal (16 CS/US pairings) v. extensive (112 CS/US pairings) Pavlovian conditioning	RMTA	Overall intake, sucrose pellet	Minimal Pavlovian conditioning is sufficient for RMTA; extensive conditioning prevents RMTA	Holland (2005)
Rat (Hooded Lister)	Lesion of Basolateral Amygdala (BLA)	RMTA	Overall Consumption, Liquid Sucrose or Maltodextrine	BLA is necessary for RMTA	Dwyer and Killcross (2006)
Rat (Long-Evans)	Neonatal ventral hippocampal lesion (NVHL)	RMTA	Overall intake, sucrose pellet	NVHL facilitates RMTA	McDannald et al. (2011)
Mouse (C57BL/6J PLC*β*1^+/−^; 129S4/SvJae PLC*β*1^+/−^)	Global PLC*β*1 knockout	RMTA	Overall intake, liquid sucrose	PLC*β*1 knockout facilitates RMTA	Kim and Koh (2016)
Mouse (C57BL/6-N)	Adolescent chronic NMDA antagonism (MK801); global administration of THC; global and hippocampal CB_1_R antagonism (Rimonabant); signal−specific inhibition of CB_1_Rs via neurosteroid (Pregnenolone; global and hippocampal); global administration of amphetamine	RMTA	Overall Consumption, Liquid Sucrose or Maltodextrine	Chronic NMDA antagonism facilitates RMTA; THC facilitates RMTA; CB_1_R antagonism blocks facilitation of RMTA by THC; pregnenolone blocks facilitation of RMTA by THC; amphetamine facilitates RMTA	Busquets-Garcia et al. (2017)
Mouse (C57BL/6-N)	Global CB1R Knockout; Global CB_1_R Antagonism (Rimonabant); Genetic Deletion of CB_1_Rs in Hippocampal GABA Neurons	RMTA	Overall Consumption, Liquid Sucrose or Maltodextrine	CB1Rs are necessary for RMTA; hippocampal CB_1_Rs are sufficient for RMTA	Busquets-Garcia et al. (2018)
Mouse (C57BL/6J)	Adolescent sub-chronic NMDA antagonism (Ketamine);	RMTA	Overall intake, liquid sucrose	Sub-chronic NMDA antagonism faciliates RMTA; risperidone blocks RMTA facilitation ketamine exposed mice	Koh et al. (2018)
Mouse (C57BL/6J)	Adolescent sub-chronic NMDA antagonism (Ketamine)	Mediated Performance	Overall intake, liquid sucrose; c-Fos mRNA (insular cortex)	Sub-chronic NMDA antagonism faciliates mediated performance; Ketamine exposed mice show increased insular cortex activity associated with facilitation of mediated performance	Wu et al. (2020)
Mouse (DN-DISC1-PrP)	Dominant-negative expression of DISC-1 gene; adolescent isolation	Mediated Performance	Mean lick cluster size; liquid sucrose; c-Fos reactivity (insular cortex)	Genetic translocation of DISC-1 facilitates RMTA; adolescent isolation in DN-DISC1-PrP mice further facilitates RMTA; haloperidol blocks facilitation of RMTA	Fry et al. (2020)

## Hallucinations and associative learning

**Fig. 1.**
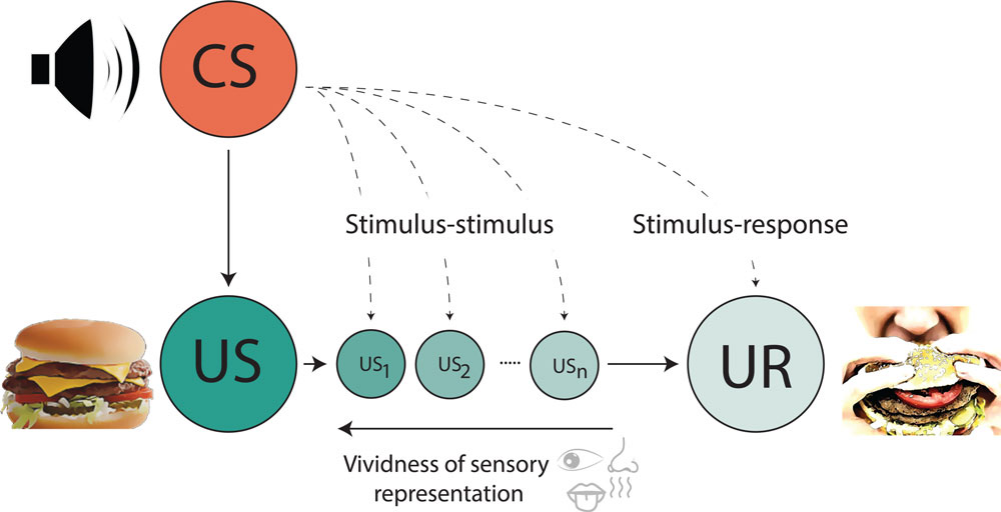
Illustration demonstrating the difference between stimulus-stimulus (S-S) and stimulus-response (S-R) aspects of learning following repeated presentation of a conditioned stimulus (CS; e.g. a tone) and unconditioned stimulus (US; e.g. palatable food). In stimulus-stimulus learning, a CS can become associated with the sensory features (e.g. taste, smell) of the US (US_1_…US_n_). By contrast, in stimulus-response learning a CS becomes directly associated with the unconditioned response (UCR; e.g. salivation and chewing). Mediated learning procedures are more consistent with an S-S account of learning, endowing the ability of the CS to evoke perceptual processing of the US – a state which appears to be both prolonged and more readily interpreted as external reality in animal models which recapitulate various aspects of schizophrenia.

The way in which an organism relates its internal states to the external world – a question crucial to understanding hallucinations – can be probed using associative learning procedures. From the perspective of associative learning, a previously neutral stimulus (e.g. tone) can readily become associated with the delivery of some biologically relevant unconditioned stimulus (US, e.g. food). After repeated pairings, the neutral stimulus, now referred to as the conditioned stimulus (CS), comes to elicit a conditioned response (CR) on its own. In Pavlov's classic example, a bell preceding the delivery of beef powder evoked salivation in his dogs (Pavlov, 1927). But what is it that drives the CR? Early psychologists suggested that the CR represented either the automatic activation of a motor response triggered by a particular stimulus (stimulus-response, S-R representations; Fig. 1), or that the CS could evoke a response via associatively-activated representations between the CS and US (stimulus-stimulus, S-S representations; Fig. 1) (Pavlov & Gantt, 1928; Rozeboom, 1958). More contemporary discussions center around the hierarchical organization of the representational structure underlying learning (Hall, 2002) and the computational rules underlying connections linking associative events (Esber & Haselgrove, 2011; Rescorla, & Wagner, 1972). Although an exhaustive discussion of these questions related to the mechanisms and contents of learning (see, Delamater & Oakeshott, 2007; Hall, 2002) are beyond the scope of this review, a venerable research history nevertheless confirms that learning mechanisms influence our perceptual experiences.

As an example of stimuli influencing perceptual processing, let us consider the smell of almonds. People typically describe almonds smelling sweet; yet there are no olfactory receptors for detection of sweetness. Rather, this perceptual experience arises through learning. Specifically, our experience of consuming food inherently involves combining gustatory and olfactory stimuli. These consistently paired experiences result in associative learning, whereby we learn to associate smell with taste, and engenders what might be considered the false perceptual experience of a sweet smell. Indeed, research has shown that when a novel scent is paired with a sweet solution, it is rated as smelling reliably sweeter than the same scent paired with water (Stevenson, Boakes, & Prescott, 1998). Here we have a clear demonstration that our perceptual experiences can be influenced by stimulus-stimulus learning, such that associatively-activated conditioned alterations in the perceptual features of a stimulus can be generated.

Discussions surrounding stimulus-stimulus associations which explicitly link the representational states observed in classical conditioning with the quasi-perceptual experience of mental imagery in humans have been addressed previously in the literature (Holland, 1990). Learning theorists have demonstrated that animals, like humans, are capable of forming perceptual representations that do not conform with reality. Holland (1981), for instance, began a series of studies that involved modifying the well-known procedure of conditioned taste aversion (CTA). Typically CTA works as follows: provide an animal access to some food item (e.g. liquid sucrose), then following consumption, administer a compound to elicit gastric malaise, such as the illness-inducing agent lithium-chloride (LiCl). Under these circumstances, the animal will consume less of the sucrose solution that was paired with LiCl, and this effect can last days or even weeks following LiCl exposure. Thus, a taste aversion has been formed on the basis of an association between the sensory features of food and gastric malaise (Garcia & Koelling, 1966; Holland, 1990). Extending these findings, Holland paired an auditory tone that had been associated with the delivery of sucrose with LiCl (Holland, 1981). Here, the idea was that because the sucrose and the LiCl were never presented together, any changes in behavior towards the sucrose must have been mediated by the ability of the tone to evoke a perceptual representation of the sucrose (think back to the scent of almonds) that was readily associable with gastric malaise produced by LiCl. This stimulus-evoked perceptual representation of a sweet taste then became associated with the LiCl-induced gastric malaise. That is, taste aversion occurred in the absence of the actual sucrose – a mental representation was sufficient. This effect came to be referred to as representation-mediated taste aversion (RMTA), and it is from this body of work that we begin to understand how environmental cues may come to elicit perceptual imagery which, under certain conditions, becomes difficult to distinguish from external reality. Later studies showed that this ability of the CS (e.g. tone) to mediate changes in behavior toward a US (e.g. sucrose) was transient in nature; mediational changes in the US could only be affected by the CS early on in the course of learning (Holland, 1998, 2005; Holland et al. 2008), suggesting that the perceptual experience evoked by the CS is more vivid and prone to error under conditions of uncertainty – that is, when less is known about a given set of environmental contingencies (but see, Holland, 2005). Thus, early on in learning, a CS appears able to evoke a realistic sensory representation of reward; however, with additional experience, the evoked representation becomes less realistic and more distinguishable from the reward. As we discuss later, in animal models of schizophrenia, these realistic sensory representations appear to dominate perceptual experiences.

Like rodents, humans are also vulnerable to the influence of associative learning procedures on perception. Whilst there is a relative dearth of experiments that have directly tested representation-mediated learning in humans (e.g., Bernstein, Laney, Morris, & Loftus, 2005), conditioned hallucinations are a related phenomenon by which a cue can come to elicit a rich perceptual experience. Although there are some nuanced differences between RMTA and conditioned hallucinations, the parallels between them offer a unique opportunity to bridge animal and human work (Corlett & Schoenbaum, 2021). In these conditioned hallucination paradigms, participants are trained to associate two sequentially presented stimuli (e.g. a light followed by a tone). Early behavioral experiments showed that, following training, the second stimulus could be perceived in its absence, contingent upon the presentation of the first stimulus – a so-called conditioned hallucination (Corn-Becker, Welch, and Fisichelli, 1949; Ellson, 1941, 1942; Leuba, 1941; Seashore, 1895; Leuba and Dunlap, 1951; Naruse and Obonai, 1953) and these early findings have been replicated using more robust psychophysiological techniques (Fishkin, 1969; Graham, 1969; Kafadar et al., 2020; Kot & Serper, 2002; Powers et al., 2017; Warburton, Wesnes, Edwards, & Larrad, 1985). Combined, these studies indicate that a person's perceptual experience can be altered through associative learning processes; however, the success of inducing conditioned hallucinations in these associative learning paradigms is highly variable. For example, while some early studies reported no evidence of a tone being able to elicit the perceptual experience of its respective, paired color (Kelly, 1934), later studies have induced at least one conditioned hallucination in up to 95% of their participants (Warburton et al., 1985) suggesting that individual and experimental factors might moderate the strength of conditioned hallucinations. In the next section, we review factors that modulate the experience of conditioned hallucinations and suggest that delineating these moderating factors may help us understand these experimental phenomena and the experience of hallucinations more broadly.

## Factors influencing the strength of conditioned hallucinations

Animal models of impaired reality testing are often conducted under conditions of food deprivation, which enhances the motivational drive of the animal along with the incentive value of the food reward. Thus, one factor that may moderate strength of conditioned hallucinations in humans is their motivation to complete the experimental task. While conditioned hallucination paradigms often use stimuli that carry no inherent motivational value, the effect of task motivation was explored by (Howells, 1944) who established a color-light pairing and informed participants that their continued employment hinged on their ability to accurately discern the hue of a colored screen in the presence of varying tones. Howells (1944) reported higher rates of conditioned hallucinations across participants than Kelly (1934), who used a similar paradigm but without the added incentive, suggesting that the motivational state of participants may moderate the effect of associative learning and attention on perception to promote the generation of conditioned hallucinations. Importantly, however, it is unclear from these experiments whether the motivational manipulation led to a true change in perception or simply a change in reporting criteria.

Further support for the role of motivational state of participants in determining the strength of conditioned hallucinations stems from examining methodological differences between studies that have reported null (Kelly, 1934) or weak results (Fishkin, 1969) as compared to those that have observed more robust effects (Powers et al., 2017). In both of the experiments that failed to report strong conditioned hallucinations, participants did not directly respond to the stimuli during training trials whereas the studies reporting more robust conditioned hallucinations required participants to make perceptual decisions about the second stimulus during training (e.g. Ellson, 1941; Howells, 1944; Powers et al., 2017). These perceptual decisions during training may have led to stronger stimulus associations (e.g. by enhancing attention to the stimuli, resulting in stronger conditioned hallucinations) and invite the suggestion that motivation and attention to contingencies may mediate the strength of conditioned hallucinations.

The associability of stimulus modalities may also affect the strength of conditioned hallucinations. Researchers have previously suggested that the success of evoking conditioned hallucinations depends upon using stimulus modalities that are readily associable (e.g. visual & auditory stimuli often co-occur; Howells, 1944). Indeed, in animals, certain stimuli are more readily associated with a response than others. For example, gastric malaise is more readily associated with food than a shock (Garcia & Koelling, 1996; Krane & Wagner, 1975). Early studies elicited conditioned hallucinations using auditory-visual (Ellson, 1941; Leuba, 1940; Naruse & Obonai, 1953), auditory-tactile (Hefferline & Perera, 1963; Leuba, 1940; Leuba & Dunlap, 1951), auditory-olfactory (Leuba, 1940; Leuba & Dunlap, 1951), visual-tactile (Leuba, 1941) and tactile-olfactory (Leuba, 1941) modality pairings. However, no study to date has directly compared the efficacy of these modalities in inducing conditioned hallucinations, with contemporary studies focusing almost exclusively on auditory-visual pairings (Graham, 1969; Kafadar et al., 2020; Kot & Serper, 2002; Powers et al., 2017; Warburton et al., 1985).

While no study has explicitly tested the effects of the stimulus-stimulus (i.e. S1-S2) modality on conditioned hallucinations, examination of related literature provides several useful predictions. Multisensory integration research has demonstrated that our perception is dominated by the most reliable senses (Eimer, 2004). For example, when speech is presented alongside incongruent visual cues (e.g. lip movements), speech perception is typically most influenced by the lip movements (Mcgurk & Macdonald, 1976), presumably because vision is considered the more reliable sense (Witten & Knudsen, 2005). That information from some senses are more reliable than others suggests that the conditionability of hallucinations may depend on the sensory modality of S1 and S2. More specifically, hallucinations may be more readily conditioned when the modality of S1 is more reliable than that of S2. In this case, information from S1 would be more likely to override the contradictory information (i.e. that S2 was absent despite the expectation formed by the repeated pairings).

Another experimental factor that appears to influence the conditionability of percepts is the use of hypnosis; a state where attention is detached from the immediate experience and becomes hyper-fixated on internal experiences, resulting in individuals being more open to suggestion (Elkins, Barabasz, Council, & Spiegel, 2015; Williamson, 2019). Many early human studies relied on hypnosis to condition hallucinations in participants (Leuba, 1941; Leuba & Dunlap, 1951; Naruse & Obonai, 1953), such that participants were inducted into a hypnotic trance before the stimulus pairings were presented. Whilst conditioned hallucinations have been elicited without hypnosis (Kafadar et al., 2020; Kot & Serper, 2002; Powers et al., 2017; Warburton et al., 1985), research suggests that hypnotic induction can strengthen conditioned hallucinations. For example, Graham (1969) tested two samples of highly suggestible participants using identical conditioning paradigms and found that conditioned hallucinations were elicited more readily in those who were hypnotized. Although the mechanism by which hypnotic trance exerts this facilitatory effect is unclear, several possibilities emerge. Hypnosis may focus attention on the stimulus-stimulus pairing, thereby enhancing the learned association (Leuba, 1941; Raz, Shapiro, Fan, & Posner, 2002). Alternatively, or in addition, hypnosis may blur the line between imagery and reality (Williamson, 2019), thereby permitting stimulus expectations, built up over repeated pairings, to unduly bias perception (Bryant & Mallard, 2003).

In summary, the strength of conditioned hallucinations appears to depend on a range of factors that include the motivational state of the participant, the attention paid to stimulus contingencies, along with the ability of participants to concentrate. These factors may be a cause or consequence of an increased tendency to recognize environmental contingencies. In this case, future studies may benefit from trying to disentangle the effects of motivation and attention on the susceptibility to conditioned hallucinations. Additionally, the importance of stimulus modalities and the mechanisms of the effect of hypnosis on conditioned hallucinations are fruitful avenues for future study.

## Computational approaches to understanding conditioned hallucinations

Associative learning generally and conditioned percepts, specifically, have been formalized using mathematical models of behavior that can be instantiated in the brain. These computational models provide a testable set of hypotheses regarding the mechanisms of conditioned hallucinations. One principle that undergirds many such models is that to infer the causes of our sensory inputs in an ever-changing and uncertain environment, expectations (e.g. predictions) are weighted against experience (e.g. sensory input). Discrepancies between predictions and input (i.e. prediction error) engender changes in expectations that better match the state of the world. This general principle describes critical associative parameters related to mediated learning and conditioned hallucinations. Within a context of associative learning, prediction errors reflect the degree to which an experienced event is anticipated and can be generally formulated by the simple equation: *λ*-V, where *λ* = maximum amount of associative strength (learning) attainable by the experienced events, and V = associative strength of factors in the context of the current experience (Schultz, 2016; Schultz & Dickinson, 2000). Large discrepancies between *λ* and V typically occur during early episodes of learning, producing prediction errors that quickly diminish as the individual anticipates future events. In this way, prediction error can inform both learning (Rescorla, & Wagner, 1972) and attention (Pearce & Hall, 1980) directed to predictive stimuli. In other words, events that do not evoke a prediction error are necessarily consistent with our current model of the world, whereas events that do evoke prediction errors lead to updating of one's expectations and world model – through learning.

In both humans and non-human animals, dopamine transients respond in a manner that reflect prediction error signaling (Nakahara, 2014; Schultz, Dayan, & Montague, 1997; Sutton & Barto, 1981). Under normal circumstances, mediated learning in animals is seen only in the early stages of learning (Holland, 1998, 2005; Holland, Lasseter, & Agarwal, 2008), when dopaminergic prediction error is expected to be high (but see, Holland, 2005). Persistent aberrant elevations in dopamine transients may lead to inappropriate attribution of value to irrelevant events, which has been posited to facilitate psychosis (Kapur, 2003; Maia & Frank, 2017). In animal models of impaired reality testing (Fry et al., 2020; Koh, Ahrens, & Gallagher, 2018; McDannald et al., 2011), early-stage processing is thought to persist (McDannald & Schoenbaum, 2009) and may be under the control of aberrant dopaminergic influences (Fry et al., 2020; Koh et al., 2018). More recent work has shown that dopamine neurons also encode detailed information about the features of a stimulus (Chang, Gardner, Di Tillio, & Schoenbaum, 2017; Takahashi et al., 2017), generating so-called ‘sensory prediction errors’ that can be used to encode and predict detailed facets of expected future events (Gardner, Schoenbaum, & Gershman, 2018). The finding that dopamine encodes specific sensory information gives credence to its role in conditioned hallucinations. Thus, prediction error offers a computational construct to inform an individual when value and sensory changes in the environment take place.

The generation of prediction error has also been emphasized through more complex models of learning such as Bayesian Inference. Bayesian Inference frameworks posit that the brain engages in statistical inference to deduce the causes of sensory information whereby beliefs and expectations (priors) are combined with sensory information (likelihood) to compute a probability (posterior), whose distribution forms the basis of perceptual inference. These prior and likelihood distributions are associated with a certain reliability or precision, which determine their contributions to the inference: a relatively more precise prior is weighted more strongly than the accompanying sensory data. In the Bayesian inference framework, a prediction error is the difference between the mean of the likelihood and prior, weighted by the relative precision of the likelihood. Thus, a new posterior can be represented as the old prior plus the prediction error.

Bayesian Inference can be implemented in the brain using a predictive coding framework – we refer to this as Bayesian predictive coding.^1^ Typically, these internal models are considered hierarchical in nature whereby sensory information begins at low levels of the hierarchy and is fed up through various layers where it is interpreted with increasing levels of abstraction. When a prediction error is generated, it is fed through each level of the hierarchy until it is resolved. Thus, predictive coding views prediction errors, such as sensory prediction errors, as the mechanism through which a person's beliefs can be updated, whereas perception is the net outcome of a weighting between priors and likelihood.

Couched within this Bayesian hierarchical predictive coding framework, recent work posits that hallucinations are the result of unduly precise priors (Corlett et al., 2019). The precision of these priors allows them to dominate incoming sensory information, thereby reconciling the prediction error caused by the discrepancy between the expectation and absence of corresponding sensory input and engendering the experience of a percept without corresponding sensory stimulation. Within this framework, conditioned hallucinations are expected to occur because the stimulus-stimulus pairing increases the precision of the prior distribution, an effect that is more profound in individuals who are prone to hallucinate. In support of this account, both treatment-seeking and non-treatment seeking samples with a history of hallucinations (Powers et al., 2017), and individuals at clinical high-risk of psychosis (Kafadar et al., 2020), show stronger conditioned hallucinations. Hierarchical Bayesian modeling of these data suggested that stronger conditioned hallucinations were driven by weighting prior beliefs more strongly than sensory evidence (Powers et al., 2017). These data suggest that individuals who are susceptible to conditioned hallucinations discount sensory information on the basis of their priors regarding the stimulus-stimulus association, thus supporting the notion that conditioned hallucinations are the result of unduly precise priors.

However, alternatives to this strong prior account of hallucinations are possible. For example, conditioned hallucinations could be the consequence of a relatively imprecise likelihood distribution. Weak sensory evidence would manifest in less precise prediction errors, which could be reconciled by perceptual priors. In the case of conditioned hallucinations, weak sensory input caused by the stimulus being presented at the perceptual threshold would lead participants to rely on prior knowledge of the associative relationship. In support of this hypothesis, reducing the precision of likelihoods has been shown to generate false percepts in hierarchical predictive models of brain function (Benrimoh, Parr, Vincent, Adams, & Friston, 2018), which is consistent with the hallucinations that emerge as a result of sensory loss (e.g. Charles Bonnet Syndrome). Furthermore, while relatively strong priors could produce hallucinations, these only occurred in contexts that also included low precision likelihoods (Benrimoh et al., 2018).

If an imprecise likelihood distribution can account for conditioned hallucinations, however, we may expect them to be accompanied by low levels of confidence, whereas conditioned hallucinations caused by strong priors should be accompanied by high confidence. Findings from Powers et al. (2017) showing that individuals who reported conditioned hallucinations did so with more confidence than those who did not hallucinate may suggest then that strong priors better account for the experience of conditioned hallucinations. However, circular inference has been demonstrated to lead to high-confidence perceptions with weak sensory evidence (Jardri, Thomas, Delmaire, Delion, & Pins, 2013; Jardri, Duverne, Litvinova, & Denève, 2017). Circular inference is a phenomenon observed within hierarchical processing systems in which impairments in signal transmission can create processing loops whereby the same signal is interpreted continuously. For example, a descending prior could be misinterpreted as an incoming sensory signal which is then propagated back up through the system. This process can be iterated many times leading to a situation in which the prior and likelihood match exactly, enabling imprecise sensory input to generate high-confidence percepts.

Thus, while the strong priors account offers one way that conditioned hallucinations could be understood, imprecise sensory input can also explain conditioned hallucinations. Resolving these two possible routes to hallucinations, within this Bayesian inference framework, is more than an academic exercise. For example, there may be subgroups of individuals who differ in the primary mechanism for their hallucinations: strong priors or imprecise sensory information. Identification of such subgroups may have implications for treatment, such that individuals with imprecise likelihood data may benefit from interventions that enhance the quality of sensory input, as in the case of Charles Bonnet syndrome where visual rehabilitation and restoring vision (e.g. via cataract surgery) has been shown to lead to a resolution of visual hallucinations (Rovner, 2006). There may also be implications for the development of hallucinations, whereby sensory abnormalities may be expected to precede (or accompany) the onset of hallucinations in individuals whose hallucinations originate via imprecise sensory input, thus presenting a marker for prevention. For example, low-level sensory impairments (e.g. photopsias) are common in the prodromal stage of schizophrenia (Silverstein, 2016) and predict later transition to psychosis (Klosterkötter, Hellmich, Steinmeyer, & Schultze-Lutter, 2001) suggesting that sensory impairments may precede the onset of clinically relevant hallucinations in some individuals. Interestingly, both these sensory abnormalities and hallucinations have been shown to remit quickly following treatment in persons with schizophrenia (Kelemen, Kiss, Benedek, & Kéri, 2013) suggesting a shared causal mechanism. Combined, these data suggests that individuals who exhibit low-grade sensory impairment may benefit from additional monitoring/treatment to reduce the risk of developing clinically-relevant hallucinations.

## Neurobiology of impaired reality testing and conditioned hallucinations

Mediated learning procedures have been utilized to study the neurobiology of impaired reality testing in rodents (McDannald & Schoenbaum, 2009) and have been employed in several animal models of schizophrenia (Fry et al., 2020; Kim & Koh, 2016; Koh et al., 2018; McDannald et al., 2011; Wu, Haberman, Gallagher, & Koh, 2020) to yield insights into the neural circuits involved in hallucination-like phenomena in animals. McDannald et al. (2011) were the first to employ mediated learning procedures in an animal model of schizophrenia. Like other animal models of schizophrenia that will be described in this review, the neonatal ventral hippocampal lesion (NVHL) model recapitulates many of the behavioral endophenotypes that have come to be agreed upon as translational correlates for cognitive, negative, and positive symptomology in animals (Tseng, Chambers, & Lipska, 2009). NVHL and control rats first received conditioning in which a light CS was paired with the delivery of sucrose pellets. Following training, rats were given injections of LiCl in the presence of the light CS, but no sucrose US was delivered. Subsequently, when provided unrestricted access to the sucrose pellets, NVHL rats consumed significantly fewer than controls. These results suggest that NVHL rats developed a stronger RMTA to sucrose, despite never being presented with sucrose in the context of illness, thus implying that illusory perceptual experiences may have been more readily conditioned in the NVHL rats. Koh et al. (2018) also reported stronger RMTA using a ketamine mouse model of schizophrenia. Furthermore, injections of the antipsychotic risperidone, shown to be effective in treating psychosis, eliminated this effect.

The molecular and signaling mechanisms underlying mediated learning have also been examined. Busquets-Garcia et al. (2017) explored the effects of Δ9-tetrahydrocannabinol (THC), the psychoactive component in cannabis, to reveal THC-induced facilitation of RMTA, which was blocked by the neurosteroid and endocannabinoid CB1R antagonist, pregnenolone. Subsequently CB1Rs in hippocampal GABAergic cells were also shown to be critical for mediated learning (Busquets-Garcia et al., 2018) and suggest these procedures can be used to model impaired reality testing following prolonged cannabis use (Ioannidou, Busquets-Garcia, Ferreira, & Marsicano, 2021). The phospholipase C *β*1 signaling pathway has also been implicated in RMTA, whereby gene-targeted knock-out of this cascade in PLC*β*1-/- mice display pronounced RMTA (Kim & Koh, 2016). Interestingly, in contrast to studies in NVHL and ketamine models wherein the mice showed stronger RMTA following minimal training, the PLC*β*1-/- animals were more vulnerable to RMTA even after receiving extensive training with light and sucrose pairings. These data are notable as RMTA is a phenomenon that typically occurs only in the early stages of learning (Holland, 1998, 2005), potentially implying that the neurophysiological abnormalities underlying schizophrenia prolong the period in which the brain conflates environmental cues with the sensory phenomena which they later come to predict, while at the same time strengthening those illusory perceptual experiences.

Alongside research on RMTA, other studies have examined related representation-mediated paradigms to study impaired-reality testing where the capacity of a CS to transfer sensory responses in the absence of a previously paired motivational US is assessed. In transgenic mice expressing a dominant-negative form of *Disrupted-in-Schizophrenia-1* (DISC-1) – a protein involved in early intracellular developmental processes – Fry et al. (2020) extensively trained mice to associate a tone (CS) with liquid sucrose (US). At test, the CS was presented, however the sucrose was replaced with unflavored water. Compared to wild-type mice, transgenic DISC-1 mice licked at the water for longer and displayed a profile of licking typically associated with consuming a sweet-tasting solution (Johnson, 2018; Johnson et al., 2010). Similar to findings from Kim and Koh (2016), these data suggest that in animal models of schizophrenia, the period over which a cue can come to evoke a sensory representation is prolonged. These effects were attenuated following antipsychotic haloperidol administration and facilitated when DISC-1 mice were exposed to early adolescent social isolation, a manipulation known to exaggerate behavioral and neurobiological phenotypes of schizophrenia in the model (Johnson et al., 2013; Niwa et al., 2013). Interestingly, DISC-1 mice showed significantly increased neural activity in the insular (gustatory) cortex following testing; suggesting disruptions in DISC-1 led to a pattern of brain activity similar to what would be expected if sucrose had actually been presented. A similar pattern of enhanced insular activity was revealed in ketamine-exposed mice that received mediated performance testing (Wu et al., 2020), whereby a sucrose solution was delivered in the presence of an almond odor. Subsequently, mice were exposed to the odor but sucrose was replaced with water. Consistent with the previously described studies in humans (Stevenson et al., 1998), the almond flavor was capable of evoking activity in putative sweet-taste responsive cells in the insular cortex. This effect was significantly augmented in ketamine-exposed mice (Wu et al., 2020), thus indicating that both auditory (Fry et al., 2020) and olfactory (Wu et al., 2020) CSs can evoke robust taste percepts in animal models of schizophrenia. This latter study also indicated that insular activity could be further enhanced by hippocampal lesions (Wu et al., 2020), which together with the necessity of CB1R in hippocampal GABAergic cells (Busquets-Garcia et al., 2018), and basolateral amygdala in representation mediated learning (Dwyer & Killcross, 2006; Johnson, Gallagher, & Holland, 2009), suggests a critical role for the limbic system in perpetuating psychotic-like behaviors in animal models. Evidence from non-human primates has also demonstrated the necessity of limbic structures in using detailed reinforcer representations to guide behavior (Baxter, Parker, Lindner, Izquierdo, & Murray, 2000; Málková, Gaffan, & Murray, 1997; West, DesJardin, Gale, & Malkova, 2011), suggesting that these results may generalize across higher-ordered mammalian species.

A handful of imaging studies have characterized the neurobiological underpinnings of conditioned hallucinations in humans. In an fMRI study, a conditioned auditory percept was accompanied by enhanced activity in supplementary auditory cortex – akin to perception of a true auditory stimulus – along with insular and anterior cingulate activation (Powers et al., 2017). A PET study of participants learning tone-visual stimulus associations found that learning was associated with changes in the interaction between the frontal and occipital regions such that, by the final scan, the presence of the auditory signal alone could activate the occipital cortex at similar levels as that of a visual stimulus (McIntosh, Cabeza, & Lobaugh, 1998). Training-related changes in the activity of prefrontal cortex activity appeared to mediate the ability of the tone to activate the occipital cortex: correlations between occipital activity and prefrontal activity changed from inhibitory to excitatory with learning. These data hint at the importance of prefrontal cortex in establishing these associations – an argument consistent with the critical role of the prefrontal cortex in establishing associations between learned events (Miller & Cohen, 2001).

## Future directions

Throughout this review, we have considered various experimental conditions under which conditioned hallucinations may arise. We have seen that the generation of conditioned hallucinations may depend on a number of factors related to the associative architecture of conditioning and computational encoding. Furthermore, we have discussed how neurobiological perturbations, which mimic physiological and pharmacological abnormalities associated with schizophrenia, increase the ease with which conditioned hallucinations can be elicited. Toward that end, we now look to create a map of what may be left to explore in regard to these phenomena.

To begin, we return to impaired reality testing in animals, and in particular, what has been learned thus far from DISC-1 mice. DISC-1 mice show an enhanced sensitivity to mediated performance (Fry et al., 2020), a scenario in which a cue comes to elicit appetitive response behaviors associated with a previously paired stimulus that is no longer present. For DISC-1 mice, it seems, the nature of these behaviors is such that they are tied to a rich internal representation of the sensorial features of the absent stimulus which are interpreted as external reality. While both humans with psychosis (Powers et al., 2017) and DISC-1 mice show an enhanced ability to represent the perceptual features of stimuli, they appear unable to use this information to adaptively guide behavior. Indeed, like humans with schizophrenia (Collins, Brown, Gold, Waltz, & Frank, 2014), the DISC-1 mouse is rigid in their behavior (Johnson et al., 2013). This counterintuitive set of findings hints at a phenotype with enhanced susceptibility to conditioned hallucinations, which warrants further exploration in humans; that is, prefrontal deficits which disrupt reward expectancy (Holland & Gallagher, 2004) when combined with increased sensory information from limbic system structures (Busquets-Garcia et al., 2018; Dwyer & Killcross, 2006) may serve to confer risk for conditioned hallucinations.

Next, the nature of conditioned hallucinations as revealed through animal studies of impaired reality testing appears quite nuanced. For example, some studies have found marked differences in the expression of these effects dependent on whether the animal learned more or less about a given associative relationship between stimuli. Specifically, associatively-conditioned cues seem to more readily evoke perceptual processing of the sensorial features of absent stimuli early on in the process of learning (Holland, 1998; Holland et al., 2008). We suggest that the neurobiological abnormalities underlying schizophrenia may prolong the period in which this enhanced susceptibility occurs – a hypothesis that could be tested in future work. Furthermore, we ask, how might the nuanced relationship between early *v.* late stages of learning relate to the idiosyncratic nature with which humans experience hallucinations? How might this influence an individual's ability to distinguish between internal and external perceptions, for instance?

Future research in this area may also aid in parsing heterogeneity in the mechanisms that subserve hallucinations among and within clinical and normative populations. More rigorously controlled studies of how different stimuli can come to elicit conditioned hallucinations, combined with careful characterization of hallucinations across individuals may be illuminating here. For example, in contrast to the hallucinations discussed throughout this paper, hallucinations experienced by individuals with psychosis spectrum disorders typically occur in the absence of an obvious, external trigger. Since strong negative emotions can increase the risk of hallucinations in people (Smith et al., 2006), it is possible that internal cues may influence hallucinations in humans. To our knowledge, the extent to which emotionally meaningful stimuli can influence the experience of conditioned hallucinations in humans has yet to be examined but would yield valuable information for understanding the clinical roots of hallucinations.

Additionally, future studies in humans should more rigorously explore the impact of motivation on conditioned hallucinations by manipulating motivation within the same study context by varying the motivator offered to participants. For example, studies could examine the extent to which monetary rewards, compared to intrinsic motivators (Morris et al., 2001), may influence the experience of conditioned hallucinations. Additionally, researchers may minimize the influence of response bias, criterion shift, and demand characteristics by using careful psychophysical techniques to assess both the presence and the strength of putative conditioned hallucinations under different motivational conditions – that is, assess stimulus discrimination rather than stimulus detection.

Finally, whilst dopamine appears to be an important modulator in these learned hallucinations, it is not yet clear whether it is the conditions for learning, or learning itself, which can lead to hallucinations. Whilst dopamine has been related to the reward-learning system (Schultz, 1998), it is also implicated in the internal pacemaker functions that allow us to maintain an internal sense of time (Buhusi & Meck, 2005) with accumulating evidence suggesting that timing and prediction error learning may be related processes (Kirkpatrick, 2014). Given that associative learning depends on a person's ability to view two independent stimuli as occurring together in time, a dysfunctional internal pacemaker could disrupt this process by leading to unrelated stimuli becoming associated. Alternatively, a dysfunctional internal pacemaker would impair a person's ability to reliably use temporal contiguities which would make them more reliant on sensory representations and previous knowledge (e.g. priors). Thus, interval timing appears integral to learning and is impaired in persons with schizophrenia (Ciullo, Spalletta, Caltagirone, Jorge, & Piras, 2016). However, the relationship, if any, between interval timing, associative learning and conditioned hallucinations is not yet clear. Future studies should therefore examine whether deficits in interval timing predict increased sensitivity to these animals, and human, paradigms.

## Conclusion

Our paper reviews data that highlights the potential utility of translational approaches for understanding hallucinations. By examining human and animal studies in unison we highlight several common factors (e.g. motivational state, stimulus-stimulus associations) which moderate the strength of conditioned hallucinations. That these paradigms reliably elicit similar experiences across species suggests a common neurobiological framework involving associative learning phenomena. Our review noted the importance of the course of learning (early *v.* late) in evoking these illusory states in animals, a parameter which has yet to be investigated in humans. Other areas of research which warrant further inquiry include the role of interval timing, influence of prefrontal cortex, hippocampal and amygdalar signaling, dopaminergic activity, and how this circuitry may relate to encoding stimuli in such a manner that predisposes individuals to hallucinations. The ability to identify and selectively isolate vulnerable circuitry would mark a step-forward in the direction of person-centered treatment and underscores the importance of increased collaboration between animal and human researchers.

## Note

1 As a note, predictive coding need not be Bayesian. Nor does Bayesian Inference depend upon a predictive coding implementation. Interested readers are directed to read Aitchison and Lengyel (2017) who provide a detailed account of how predictive coding does, and does not, relate to Bayesian Inference.
